# Comparison of the Repeatability of Macular Vascular Density Measurements Using Four Optical Coherence Tomography Angiography Systems

**DOI:** 10.1155/2019/4372580

**Published:** 2019-11-23

**Authors:** Jingyuan Yang, Mingzhen Yuan, Erqian Wang, Youxin Chen

**Affiliations:** Department of Ophthalmology, Peking Union Medical College Hospital, Chinese Academy of Medical Sciences, Beijing 100730, China

## Abstract

The aim of this study was to compare the repeatability of optical coherence tomography angiography (OCT-A) measurements of macular vessel density using four OCT-A systems, including Heidelberg Spectralis HRA, Optovue RTVue XR, Zeiss Cirrus HD-OCT 5000, and Topcon DRI OCT Triton. A cross-sectional design was used for this study. The vascular density and vascular length density of the superficial and deep retinal capillary plexuses were imaged with OCT-A using 3 mm and 6 mm scan patterns and were calculated using ImageJ. Comparisons of intraclass correlation coefficients (ICC) were conducted. We found that the OCT-A systems had various levels of repeatability. Zeiss had better repeatability for vessel density than the other systems (overall ICC = 0.936). Optovue had better repeatability for vessel length density when the 6 mm scan pattern was used (ICC = 0.680 and 0.700 for retinal superficial and deep capillary plexus, respectively). We concluded that repeatability varied when different scan patterns of various OCT-A systems were used for imaging the superficial retinal and deep capillary plexuses. Results should be seen as valid only for a given method. The repeatability of various OCT-A systems should be considered in clinical practice and in clinical trials that use OCT-A metrics as outcome measures.

## 1. Introduction

Optical coherence tomography angiography (OCT-A) was recently developed for imaging the retinal vasculature without dye injection [1]. OCT-A allows for the measurement of both the superficial and the deep retinal vessels quantitatively, including those in the macular region. Vessel density, which is quantified by calculating the percentage of the OCT image occupied by blood flow information as a fraction of the total image area, and vessel length density, which is quantified by calculating the percentage of skeletonized images of the retinal vasculature occupied by the vascular skeleton as a fraction of the total image area, have gained increasing popularity and represent promising quantitative metrics for future studies.

With OCT-A quantification becoming increasingly common, there is a pressing need to understand whether OCT-A systems can provide reliable and stable quantitative results and to determine which OCT-A system has better repeatability when using a specific scan pattern in clinical practice. Several studies have evaluated the reproducibility of various OCT-A systems; the results of these studies indicate that the outcomes of these instruments are generally uninterchangeable [2–4]. Therefore, the repeatability of one OCT-A system should be taken into consideration when that OCT-A system is used. However, none of these aforementioned studies compared the repeatability of macular vessel density measurements taken with different OCT-A systems in detail.

Furthermore, assessment of various retinal diseases requires different scan areas and different OCT-A image resolutions. In the available literature that examined the validity and repeatability of these systems, emphasis was placed on using one pattern for a scan area, ignoring the use of multiple patterns for the scan area. Given the popularity of OCT-A, a more recent trend is to perform OCT-A examination with a larger scan area, as this provides a more relevant assessment of traditional fluorescence angiography. Evaluation of the repeatability of various scan areas is needed for future application of OCT-A.

Therefore, there is a need to investigate the repeatability of OCT-A systems using various scan patterns. If differences in the repeatability of macular vascular measurements exist between various OCT-A systems, this would be consequential for any clinical trial or research that uses a specific OCT-A system. To the best of our knowledge, no published study has compared the repeatability of various OCT-A systems for various scan areas and different retinal capillary layers. This study examines the repeatability of macular vascular density measurements calculated with four commercially available OCT-A devices.

## 2. Materials and Methods

### 2.1. Participants

This cross-sectional study was approved by the Peking Union Medical College Hospital Institutional Review Board, Beijing, China (reference number: ZS-1976). All procedures followed the tenets of the Declaration of Helsinki. The 48 adult subjects (13 males and 35 females) included in this study were outpatients recruited from the Peking Union Medical College Hospital between April 2019 and May 2019; individual participants could not be identified using the collected data. The inclusion criteria required each subject to be a consenting adult aged 18 and over, with healthy eyes. The exclusion criteria included a history of retinal and choroidal diseases, glaucoma, any other ocular diseases, and previous ocular surgery.

The subjects underwent a complete ophthalmic examination, including measurement of best-corrected visual acuity and refractive errors, slit lamp biomicroscopy, fundoscopy, and OCT-A, along with documentation of a complete medical history.

### 2.2. Optical Coherence Tomography Angiography Acquisition and Processing

All subjects underwent OCT-A imaging with Heidelberg Spectralis HRA (Heidelberg Engineering GmbH, Germany), Optovue RTVue XR (Optovue, Inc., USA), Zeiss Cirrus HD-OCT 5000 (Carl Zeiss Meditec, Inc., USA), and Topcon DRI OCT Triton (Topcon, Corp., Japan) in the same clinical setting. The four OCT-A examinations were performed twice by one experienced ophthalmologist in dark rooms on the same day, and subjects were blinded to personal information during and after the study. There was sufficient interval for rest between the OCT-A examinations carried out with the four systems, but the interval between the two examinations for each system was as short as possible to minimize the influence of diurnal physiological conditions [5]. The pupil was not dilated before examination, and an internal fixation target in the OCT device was used. The image quality scores of the included OCT-A images acquired with the Optovue and Zeiss devices were not less than nine, and these scores were generated by the devices automatically. The quality of the OCT-A images acquired with the Heidelberg and Topcon devices was evaluated by two retinal specialists independently (Jingyuan Yang and Mingzhen Yuan,). In cases of disagreement, a third retinal specialist (Youxin Chen) made the final decision. Unacceptable images, such as those with poor scan quality or those not centered on the fovea, were excluded [6]. The included OCT-A images were exported using Heidelberg Eye Version 1.10.2.0 (Heidelberg), AngioVue Version 2017.1.0.155 (Optovue), Zeiss inbuilt software Version 9.5.2.19038 (Zeiss) (analyzed with Version 10.0.0.14618), and IMAGEnet 6 Version 1.1.4 (Topcon). The 3 × 3 mm (3 mm scan pattern) and 6 × 6 mm (6 mm scan pattern) scan patterns were used with maximal resolution. The scan protocol for the 3 × 3 mm scan pattern composed of 512 × 512 scans for Heidelberg, 304 × 304 scans for Optovue, 429 × 429 scans for Zeiss, and 320 × 320 scans for Topcon; the scan protocol for the 6 × 6 mm scan pattern was composed of 512 × 512 scans for Heidelberg, 400 × 400 scans for Optovue, 429 × 429 scans for Zeiss, and 512 × 512 scans for Topcon. Default segmentation was not adjusted and manual correction of segmentation was not applied. The retinal capillary plexus was divided into the superficial capillary plexus (SCP) and the deep capillary plexus (DCP). Default brightness and contrast were not adjusted to avoid manual bias. Considering that only two of these OCT-A systems provided personalized quantitative measures and to ensure that the OCT-A images were compared in the same setting, we used the third-party software ImageJ v2.0.0 (National Institutes of Health, available at https://imagej.net/Fiji/Downloads) to analyze quantitative metrics. To investigate the reliability of the third-party software, a comparison of the repeatability of the quantitative metrics generated by the inbuilt software of the OCT-A systems and the third-party software was conducted first, in which the original vessel length density was converted into a decimal format. The macular OCT-A images were binarized with the Phansalkar local binarization thresholding method, which has been used on en face OCT-A images in recent studies [7–10]. The default parameter of a radius of 15 pixels in ImageJ was used. Once each pixel in the en face OCT-A image was determined to represent flow or nonflow using the thresholding method, binarized images were generated for further analysis, including analysis of vessel density and generation of skeletonized images for analyzing vessel length density. Binarized images were then skeletonized using ImageJ. Vessel density [11, 12] and vessel length density [13, 14] were subsequently calculated.

### 2.3. Statistical Analysis

All statistical analyses were performed using SPSS version 25.0 (IBM Corp., USA). Data are presented as mean ± standard deviation (SD) or 95% confident interval (CI). The repeatability of the two consecutive measurements was assessed using the intraclass correlation coefficient (ICC). The ICC was used to determine the repeatability for each system by employing a two-way random effects model. The degree of repeatability was classified according to the ICC as follows: slight (0–0.2), fair (0.21–0.4), moderate (0.41–0.6), substantial (0.61–0.8), and almost perfect (0.8–1) [15]. To determine the intended sample size, the minimum acceptable repeatability (ICC) was set at 0.6, and the significance level and power was set at 0.05 and 0.8, respectively. There was no dropout subject. The values of expected repeatability (ICC) for vessel density and vessel length density were set according to the overall results generated by the inbuilt software of the Optovue (0.808 for vessel density) and Zeiss (0.858 for vessel density and 0.973 for vessel length density) systems, which were the only two OCT-A systems that provided quantification functions. The minimum sample size should be 44 for vessel density and 5 for vessel length density, and the sample size of the present study met this requirement [16]. To compare the ICC values for intradevice repeatability between two OCT-A systems, we used the statistical method of Diedenhofen and Musch [17]. Statistical significance was defined as *P* < 0.05.

## 3. Results

Ninety-six eyes from 48 subjects (13 males and 35 females) were included. The mean (SD) age was 30.69 ± 4.67 years. The mean (SD) vessel density and vessel length density values measured with the four systems are summarized in [Supplementary-material supplementary-material-1]; there were no missing data to report. No obvious segmentation errors were noticed.

The ICC values of the measurements derived with the built-in software of the Optovue and Zeiss systems and the third-party software of each system are summarized in Tables 1 and 2, respectively. The comparison of repeatability of the measurements derived with the built-in software and the third-party software is shown in Table 3. No significant difference in ICC values was noticed except for the ICC of the vessel density in the SCP imaged with the 6 mm scan pattern using Zeiss; this suggests that the third-party software showed comparable or even better repeatability than the built-in software during analysis.

When using the third-party software, the mean ICC for the measurements taken by each OCT-A system was 0.652, 0.881, 0.658, and 0.936 for vessel density, and 0.577, 0.925, 0.626, and 0.979 when using Heidelberg, Optovue, Topcon, and Zeiss, respectively. The comparison of the repeatability of each system is summarized in Table 4.

### 3.1. Vessel Density

The ICCs of the vessel density measurements generated using both the 3 mm and 6 mm scan patterns in Zeiss exceeded 0.6, and the ICCs for the measurements derived using the 6 mm scan pattern in Optovue exceeded 0.6. The ICCs of the vessel density measurements of the SCP layer obtained using the 3 mm scan pattern in Heidelberg and Topcon exceeded 0.6 as well. The ICC for the vessel density of the DCP layer obtained using the 3 mm scan pattern in Zeiss was higher than that generated using Optovue (*P*=0.0065). The ICCs for the vessel density of the DCP layer generated using the 3 mm scan pattern and the ICCs for the vessel density measurements of both the SCP and DCP layers derived using the 6 mm scan pattern in Zeiss were significantly higher than the ICCs of the measurements generated by both Heidelberg and Topcon (all *P* values <0.05). The ICCs of the vessel density measurements generated using the 6 mm scan pattern in Optovue were higher than the ICC of the vessel density measurement of the SCP layer derived using the 6 mm scan pattern in Heidelberg and the ICC of the vessel density of the DCP layer derived using the 6 mm scan pattern in Topcon (*P*=0.0023 and 0.0009, respectively). No significant differences in ICCs were noticed between Heidelberg and Topcon (all *P* values >0.05).

### 3.2. Vessel Length Density

The ICCs for vessel length density measurements for both the SCP and DCP layers obtained using the 6 mm scan pattern in Optovue exceeded 0.6. The ICC of the vessel length density measurement of the DCP layer derived using the 3 mm scan pattern in Zeiss exceeded 0.6 but was close to 0.6 for the vessel length density measurements of the SCP layer. However, no significant differences were observed between the ICCs of the vessel length density measurements derived with Optovue and Zeiss (all *P* values >0.05). The ICCs of the vessel length density of the SCP layer obtained using the 3 mm scan pattern in both Heidelberg and Topcon exceeded 0.6. When using Optovue, the ICCs of the vessel length densities of both the SCP and DCP layers derived using the 6 mm scan pattern were higher than the ICCs of those generated using Heidelberg, and the ICCs of the vessel length density of the SCP layer obtained with using 3 mm scan pattern and the DCP layer measurements derived with the 6 mm scan pattern in Optovue were higher than the ICCs of those generated using Topcon (all *P* values <0.05). When using Zeiss, the ICC of the vessel length density measurement of the SCP layer derived using the 3 mm scan pattern was higher than the ICC of that generated using Topcon, and the ICC of the vessel length density of the DCP layer derived using the 6 mm scan pattern was higher than the ICC of that generated using both Heidelberg and Topcon (all *P* values <0.05).

## 4. Discussion

In the present study, we compared the repeatability of four OCT-A systems. Each system was evaluated using the ICCs for the macular vessel density and vessel length density measurements of both the SCP and DCP layers derived using 3 mm and 6 mm scan patterns. We found that various OCT-A systems showed different levels of repeatability, and no OCT-A system showed practical repeatability in all conditions. Zeiss had better repeatability for measurement of vessel density than the other systems. Optovue had better repeatability for measurement of vessel length density using the 6 mm scan pattern. The repeatability of OCT-A systems should be considered in clinical practice, research, and clinical trial.

To investigate whether the third-party software could provide acceptable results, we compared the third-party software and the commercial built-in software of the OCT-A systems that provided quantification functions first; we found that the third-party software showed comparable repeatability with the built-in software in most cases when quantifying OCT-A images (Table 3). Since the OCT-A images of one eye are supposed to have similar vessel density and vessel length density values, the third-party software showed better repeatability when analyzing vessel density in the SCP layer with the 6 mm scan pattern in Zeiss. Therefore, we believed that using the third-party software could provide reliable results.

The repeatability between scans on individual commercialized OCT-A instruments, including Optovue, Topcon, and Zeiss has been reported to be high [5, 18–23]. Nevertheless, the different evaluation approaches used in these studies and in the present study (especially the thresholding methods and the evaluated regions) make it almost impossible to conduct comparisons among various studies, although these aforementioned studies reported that these devices showed good or even excellent repeatability. Additionally, a few previous studies had demonstrated that the quantitative measurements were not directly interchangeable among different OCT-A systems [2–4]. However, only few studies compared their repeatability on the same cohort. It is essential to perform OCT-A examinations using a reliable and repeatable system for clinical diagnosis and follow-up. The results of our study provide real-world data for improved examination in clinical practice.

In the present study, we used methods that previous studies had applied to evaluate OCT-A images. The metrics of vessel density and vessel length density were widely used in previous studies to evaluate retinal microvasculature quantitatively [11–14], and they were also calculated by the inbuilt software of Optovue and Zeiss, respectively. In the present study, the Heidelberg and Topcon systems did not provide inbuilt software for quantitative analysis. To calculate the metrics, an appropriate thresholding method for image binarization is essential. Rabiolo et al. [24] compared seven thresholding methods, and only one local thresholding method was included. However, in clinical practice, local threshold methods produce a binarized image that appears more uniform because these methods account for local variations in image appearance. The Phansalkar local thresholding method was used on OCT-A images in a recent study [7–10]. This thresholding method was proposed to address the nonuniform appearance of images [25]. In our study, the Phansalkar thresholding method enabled us to distinguish subtle capillary networks, which is believed to generate more accurate binarized images compared with ground truth. Nevertheless, given the lack of a standard method of assessing the quality of binarization thresholding methods, it is difficult to conclude that any one method is superior to the others.

Furthermore, we evaluated the SCP and DCP layers using 3 mm and 6 mm scan patterns. The OCT-A images of the DCP were obtained using different OCT-A systems with different projection artifact removal algorithms and light sources of different wavelengths [26, 27]. Therefore, the repeatability of OCT-A images of the SCP and DCP were supposed to be evaluated separately. On the other hand, the OCT-A images of 3 mm and 6 mm scan patterns had different resolutions and scan areas, which suggested that OCT-A images of different scan areas should be evaluated separately.

None of these OCT-A systems had the best repeatability in all circumstances (Tables 2 and 4). Heidelberg had a relatively poor repeatability in our study. The main explanation may be the long acquisition time and bright fixation target, which could lead to more motion artifacts and eye blinks [28]. Topcon had a poor repeatability in the measurement of the vascular density of the DCP; notably, the Topcon system was the only swept-source OCT used in the present study and should have had better performance on deep tissue than spectral-domain OCT. We speculate that its projection artifact removal algorithm and motion artifact correction algorithm may need further development. Among the four OCT-A systems, Optovue and Zeiss had inbuilt software for quantitative analysis in sequence; not surprisingly, they had better repeatability for quantitative analysis. Interestingly, Optovue had better repeatability when the 6 mm scan pattern was used. One of the possible reasons is that the 6 mm scan pattern covers a larger area, so that local differences in OCT-A images had less influence on the vessel density and vessel length density values. The ICC values in the present study may differ from those of previous studies; this may be because we recruited subjects from a real clinical practice rather than from experienced staff, and the methods we used were not completely similar to those of these previous studies.

However, there were several limitations in our study. Firstly, we enrolled only healthy eyes; further studies including larger numbers of patients are needed to confirm our present findings. On the other hand, this study was not influenced by obvious segmentation errors because no eyes with retinal diseases were enrolled [29]. Further investigation is needed to evaluate the influence of segmentation approaches on the repeatability of these OCT-A systems. Secondly, the majority of the subjects were young adults hence we could not evaluate the influence of age on the measurements of each device; on the contrary, elderly subjects, especially those with macular diseases, who may be not be compliant and cooperative enough during the examination and may lead to poor OCTA image quality and motion artifacts, were not enrolled in the present study [30]. Although evaluation of the repeatability of OCT-A devices with elderly eyes may lead to unstandardized evaluation methods, OCT-A examination in clinical practice is usually performed on elderly patients. Therefore, further studies focusing on subjects of less compliance are needed to evaluate the repeatability of OCT-A systems. Additionally, whether age has an influence on the repeatability of quantification of the macular capillary is unknown; this needs further investigation as well [31]. More importantly, although our results were valid for the given methodology, updates of OCT-A software and alterations of methods may affect the results.

## 5. Conclusions

All the OCT-A systems used in the present study may provide images with sufficient quality for qualitative analysis, but not all of the systems showed good repeatability for quantitative analysis of macular vessels. The present study provided detailed comparisons of the repeatability of various OCT-A systems with different scan patterns. The repeatability of various OCT-A systems should be considered when using a specific scan pattern for quantitative assessment in clinical practice. Perhaps most importantly, this study suggests that ICC values should be seen as valid only for a given methodology. This has significant consequences for clinical trials involving the use of vessel density and vessel length density values as outcome measures. Future studies comparing the repeatability of each OCT-A system in eyes with retinal diseases would be worthwhile.

## Figures and Tables

**Table 1:** Intraclass correlation coefficients (95% confidence interval) of the vessel density and vessel length density measurements derived with the built-in software of the Optovue and Zeiss systems.

System	Vessel density				Vessel length density			
	3 mm scan pattern		6 mm scan pattern		3 mm scan pattern		6 mm scan pattern	
	SCP	DCP	SCP	DCP	SCP	DCP	SCP	DCP
Optovue	0.579 (0.429–0.698)	0.462 (0.289–0.606)	0.740 (0.634–0.819)	0.718 (0.605–0.802)	—	—	—	—
Zeiss	0.682 (0.559–0.776)	—	0.316 (0.124–0.485)	—	0.705 (0.588–0.793)	—	0.358 (0.171–0.521)	—

DCP: deep capillary plexus; SCP: superficial capillary plexus.

**Table 2:** Intraclass correlation coefficients (95% confidence interval) of the vessel density and vessel length density measurements derived with the four optical coherence tomography angiography systems using third-party software.

System	Vascular density				Vascular length density			
	3 mm scan pattern		6 mm scan pattern		3 mm scan pattern		6 mm scan pattern	
	SCP	DCP	SCP	DCP	SCP	DCP	SCP	DCP
Heidelberg	0.623 (0.484–0.732)	0.465 (0.292–0.608)	0.278 (0.083–0.453)	0.407 (0.226–0.561)	0.683 (0.559–0.777)	0.483 (0.313–0.622)	0.218 (0.020–0.401)	0.408 (0.227–0.562)
Optovue	0.598 (0.452–0.713)	0.450 (0.275–0.596)	0.730 (0.621–0.811)	0.670 (0.543–0.767)	0.477 (0.307–0.618)	0.577 (0.427–0.697)	0.680 (0.556–0.775)	0.700 (0.581–0.789)
Topcon	0.675 (0.549–0.771)	0.260 (0.064–0.437)	0.497 (0.331–0.634)	0.108 (−0.094–0.301)	0.736 (0.629–0.816)	0.281 (0.087–0.455)	0.637 (0.501–0.742)	0.107 (−0.094–0.300)
Zeiss	0.732 (0.624–0.813)	0.785 (0.694–0.851)	0.773 (0.678–0.842)	0.769 (0.672–0.839)	0.598 (0.453–0.713)	0.730 (0.621–0.812)	0.529 (0.369–0.659)	0.706 (0.589–0.794)

DCP: deep capillary plexus; SCP: superficial capillary plexus.

**Table 3:** *P* values for comparison of the intraclass correlation coefficients of the vascular density and vascular length density measurements derived with the built-in software of the optical coherence tomography angiography systems and third-party software.

OCT-A systems	3 mm scan pattern		6 mm scan pattern	
	SCP	DCP	SCP	DCP
Optovue (vessel density)	0.8429	0.9177	0.8821	0.5270
Zeiss (vessel density)	0.4945	—	<0.0001	—
Zeiss (vessel length density)	0.2019	—	0.1442	—

DCP: deep capillary plexus; SCP: superficial capillary plexus.

**Table 4:** *P* values for comparison of the intraclass correlation coefficients of vessel density and vessel length density measurements derived with the four optical coherence tomography angiography systems.

System 1	System 2	Vascular density				Vascular length density			
		3 mm scan pattern		6 mm scan pattern		3 mm scan pattern		6 mm scan pattern	
		SCP	DCP	SCP	DCP	SCP	DCP	SCP	DCP
Heidelberg	Optovue	0.8500	0.9283	0.0023	0.0724	0.1344	0.5341	0.0040	0.0395
Heidelberg	Topcon	0.6695	0.2598	0.2178	0.1248	0.6118	0.2587	0.0117	0.1223
Heidelberg	Zeiss	0.3353	0.0085	0.0004	0.0055	0.4925	0.0566	0.0815	0.0344
Optovue	Topcon	0.5380	0.2998	0.0690	0.0009	0.0450	0.0799	0.7185	0.0003
Optovue	Zeiss	0.2491	0.0065	0.6386	0.3258	0.4175	0.1990	0.2542	0.9551
Topcon	Zeiss	0.5915	0.0002	0.0221	<0.0001	0.2326	0.0024	0.4356	0.0003

DCP: deep capillary plexus; SCP: superficial capillary plexus.

## Data Availability

The data used to support the findings of this study are available from the corresponding author upon request.

## Supplementary Materials

Supplementary Materials Table S1 shows the mean (standard deviation) vessel density and vessel length density values measured with the four optical coherence tomography angiography systems using third-party software.

## References

[1] Spaide R. F., Fujimoto J. G., Waheed N. K., Sadda S. R., Staurenghi G. (2018). Optical coherence tomography angiography. *Progress in Retinal and Eye Research*.

[2] Munk M. R., Giannakaki-Zimmermann H., Berger L. (2017). OCT-angiography: a qualitative and quantitative comparison of 4 OCT-A devices. *PLoS One*.

[3] Mihailovic N., Brand C., Lahme L. (2018). Repeatability, reproducibility and agreement of foveal avascular zone measurements using three different optical coherence tomography angiography devices. *PLoS One*.

[4] Li X. X., Wu W., Zhou H. (2018). A quantitative comparison of five optical coherence tomography angiography systems in clinical performance. *International Journal of Ophthalmology*.

[5] Yanik Odabas O., Demirel S., Ozmert E., Batioglu F. (2018). Repeatability of automated vessel density and superficial and deep foveal avascular zone area measurements using optical coherence tomography angiography: diurnal findings. *Retina*.

[6] Al-Sheikh M., Ghasemi Falavarjani K., Akil H., Sadda S. R. (2017). Impact of image quality on OCT angiography based quantitative measurements. *International Journal of Retina and Vitreous*.

[7] Mehta N., Liu K., Alibhai A. Y. (2019). Impact of binarization thresholding and brightness/contrast adjustment methodology on optical coherence tomography angiography image quantification. *American Journal of Ophthalmology*.

[8] Spaide R. F. (2016). Choriocapillaris flow features follow a power law distribution: implications for characterization and mechanisms of disease progression. *American Journal of Ophthalmology*.

[9] Tang F. Y., Ng D. S., Lam A. (2017). Determinants of quantitative optical coherence tomography angiography metrics in patients with diabetes. *Scientific Reports*.

[10] Uji A., Balasubramanian S., Lei J., Baghdasaryan E., Al-Sheikh M., Sadda S. R. (2017). Choriocapillaris imaging using multiple en face optical coherence tomography angiography image averaging. *JAMA Ophthalmology*.

[11] Jia Y., Tan O., Tokayer J. (2012). Split-spectrum amplitude-decorrelation angiography with optical coherence tomography. *Optics Express*.

[12] Jia Y., Morrison J. C., Tokayer J. (2012). Quantitative OCT angiography of optic nerve head blood flow. *Biomedical Optics Express*.

[13] Chu Z., Lin J., Gao C. (2016). Quantitative assessment of the retinal microvasculature using optical coherence tomography angiography. *Journal of Biomedical Optics*.

[14] Reif R., Qin J., An L., Zhi Z., Dziennis S., Wang R. (2012). Quantifying optical microangiography images obtained from a spectral domain optical coherence tomography system. *International Journal of Biomedical Imaging*.

[15] Landis J. R., Koch G. G. (1977). The measurement of observer agreement for categorical data. *Biometrics*.

[16] Walter S. D., Eliasziw M., Donner A. (1998). Sample size and optimal designs for reliability studies. *Statistics in Medicine*.

[17] Diedenhofen B., Musch J. (2015). cocor: a comprehensive solution for the statistical comparison of correlations. *PLoS One*.

[18] Chen C.-L., Bojikian K. D., Xin C. (2016). Repeatability and reproducibility of optic nerve head perfusion measurements using optical coherence tomography angiography. *Journal of Biomedical Optics*.

[19] Lei J., Durbin M. K., Shi Y. (2017). Repeatability and reproducibility of superficial macular retinal vessel density measurements using optical coherence tomography angiography en face images. *JAMA Ophthalmology*.

[20] Anegondi N., Kshirsagar A., Mochi T. B., Sinha Roy A. (2018). Quantitative comparison of retinal vascular features in optical coherence tomography angiography images from three different devices. *Ophthalmic Surgery, Lasers and Imaging Retina*.

[21] Al-Sheikh M., Tepelus T. C., Nazikyan T., Sadda S. R. (2017). Repeatability of automated vessel density measurements using optical coherence tomography angiography. *British Journal of Ophthalmology*.

[22] You Q., Freeman W. R., Weinreb R. N. (2017). Reproducibility of vessel density measurement with optical coherence tomography angiography in eyes with and without retinopathy. *Retina*.

[23] Manalastas P. I. C., Zangwill L. M., Saunders L. J. (2017). Reproducibility of optical coherence tomography angiography macular and optic nerve head vascular density in glaucoma and healthy eyes. *Journal of Glaucoma*.

[24] Rabiolo A., Gelormini F., Sacconi R. (2018). Comparison of methods to quantify macular and peripapillary vessel density in optical coherence tomography angiography. *PLoS One*.

[25] Phansalkar N., More S., Sabale A., Joshi M. Adaptive local thresholding for detection of nuclei in diversity stained cytology images.

[26] Zhang M., Hwang T. S., Campbell J. P. (2016). Projection-resolved optical coherence tomographic angiography. *Biomedical Optics Express*.

[27] Zhang A., Zhang Q., Wang R. K. (2015). Minimizing projection artifacts for accurate presentation of choroidal neovascularization in OCT micro-angiography. *Biomedical Optics Express*.

[28] Lei J., Pei C., Wen C., Abdelfattah N. S. (2018). Repeatability and reproducibility of quantification of superficial peri-papillary capillaries by four different optical coherence tomography angiography devices. *Scientific Reports*.

[29] Lauermann J. L., Woetzel A. K., Treder M. (2018). Prevalences of segmentation errors and motion artifacts in OCT-angiography differ among retinal diseases. *Graefe’s Archive for Clinical and Experimental Ophthalmology = Albrecht von Graefes Archiv fur klinische und experimentelle Ophthalmologie*.

[30] Lim H. B., Kim Y. W., Kim J. M., Jo Y. J., Kim J. Y. (2018). The importance of signal strength in quantitative assessment of retinal vessel density using optical coherence tomography angiography. *Scientific Reports*.

[31] Jo Y. H., Sung K. R., Shin J. W. (2019). Effects of age on peripapillary and macular vessel density determined using optical coherence tomography angiography in healthy eyes. *Investigative Opthalmology & Visual Science*.

