# Supplementary material for: Comparison of the Repeatability of Macular Vascular Density Measurements Using Four Optical Coherence Tomography Angiography Systems
Source: J Ophthalmol. 2019 Nov 23;2019:4372580. doi: 10.1155/2019/4372580 (PMC6906812; doi:10.1155/2019/4372580)
Supplement: Supplementary Materials — Table S1 shows the mean (standard deviation) vessel density and vessel length density values measured with the four optical coherence tomography angiography systems using third-party software. [file 4372580.f1.docx]

## Supplemental Table

**Table S1** shows the mean (standard deviation) vessel density and vessel length density values measured with the four optical coherence tomography angiography systems using third-party software.

| Scan pattern | Layer | Vascular density | | | | Vascular length density | | | |
| --- | --- | --- | --- | --- | --- | --- | --- | --- | --- |
|  |  | Heidelberg | Optovue | Topcon | Zeiss | Heidelberg | Optovue | Topcon | Zeiss |
| 3 mm scan pattern | SCP | 0.39 (0.03) | 0.39 (0.02) | 0.48 (0.02) | 0.44 (0.03) | 0.17 (0.01) | 0.19 (0.01) | 0.24 (0.01) | 0.15 (0.01) |
|  | DCP | 0.40 (0.03) | 0.47 (0.02) | 0.45 (0.03) | 0.40 (0.04) | 0.20 (0.01) | 0.27 (0.01) | 0.25 (0.01) | 0.14 (0.01) |
| 6 mm scan pattern | SCP | 0.39 (0.04) | 0.36 (0.03) | 0.49 (0.01) | 0.55 (0.03) | 0.20 (0.02) | 0.19 (0.02) | 0.24 (0.01) | 0.22 (0.01) |
|  | DCP | 0.32 (0.05) | 0.38 (0.04) | 0.44 (0.02) | 0.49 (0.04) | 0.20 (0.03) | 0.22 (0.03) | 0.26 (0.01) | 0.23 (0.01) |

DCP: deep capillary plexus; SCP: superficial capillary plexus
